# Patterns of Management of Patients With Dual Disorder (Psychosis) in Italy: A Survey of Psychiatrists and Other Physicians Focusing on Clinical Practice

**DOI:** 10.3389/fpsyt.2018.00575

**Published:** 2018-11-13

**Authors:** Massimo Clerici, Andrea de Bartolomeis, Sergio De Filippis, Giuseppe Ducci, Icro Maremmani, Giovanni Martinotti, Fabrizio Schifano

**Affiliations:** ^1^School of Medicine and Surgery-University of Milano Bicocca, Milan, Italy; ^2^Psychiatric Department, Azienda Socio Sanitaria Territoriale (ASST) di Monza, Monza, Italy; ^3^Section of Psychiatry, Department of Neuroscience, Reproductive Sciences and Odontostomatology, University of Naples Federico II, Naples, Italy; ^4^Department of Neuropsychiatry, Villa von Siebenthal Neuropsychiatric Hospital and Clinic, Genzano di Roma, Italy; ^5^Mental Health Department, Azienda Sanitaria Locale Roma 1, Rome, Italy; ^6^Santa Chiara University Hospital, University of Pisa, Pisa, Italy; ^7^Department of Neuroscience, Imaging, and Clinical Sciences, University “G.d'Annunzio” Chieti-Pescara, Chieti, Italy; ^8^Psychopharmacology, Drug Misuse and Novel Psychoactive Substances Research Unit, School of Life and Medical Sciences, University of Hertfordshire, Hatfield, United Kingdom

**Keywords:** schizophrenia, psychosis, psychotic disorder, substance use disorder, survey, management, dual disorder

## Abstract

Patients with severe psychotic disorders such as schizophrenia, schizoaffective, and bipolar disorders frequently suffer from concomitant substance use disorders (SUDs)–Dual Disorder (DD) patients. In order to better understand current practices for management of patients with psychotic episodes and concomitant SUD in Italy, we carried out a survey of psychiatrists on current routine practice among prescribers. These aspects can help to identify at-risk patients, improve current prescribing practices, and favor early intervention. An *ad hoc* survey of 17 questions was administered to psychiatrists via electronic polling and on-line distribution; 448 completed questionnaires were collected. Comorbid substance abuse was most frequently diagnosed within the context of anxiety disorder (46%), followed by bipolar disorder (25%), and schizophrenia/schizoaffective disorder (12%). The vast majority of respondents felt that patient management was becoming more complex due to substance abuse. The areas reported to be most affected in patients with SUD were functioning, interpersonal relations, and impulsivity, while sensory perception disorders, ideation, agitation, and impulsivity were the most frequently reported symptoms. In the acute setting, haloperidol was used as the first-line agent of choice followed by aripiprazole and olanzapine. In the maintenance phase, aripiprazole was the dominantly used first-line agent, followed by olanzapine. Almost half of respondents used long-acting agents, while about one-third did not. Among those prescribing long-acting agents, efficacy, control of impulsivity, and control of specific symptoms were cited as motivators, while in the maintenance phase, better adherence, and tolerability were mainly cited. From the responses to the present survey, it is clear that the respondents are aware of the problem of SUD in psychotic patients. While treatment be optimized in terms of the choice and formulation of antipsychotics, greater emphasis should be placed on efficacy, tolerability, and the negative metabolic consequences of some antipsychotics. When considering the ideal antipsychotic, long-acting agents were considered to be superior in reducing relapse, even if current treatment guidelines often give preference to oral formulations.

## Introduction

Patients affected by severe psychiatric disorders such as schizophrenia, schizoaffective, and bipolar disorders, with psychotic features, frequently suffer from concomitant substance use disorders (SUDs)–Dual Disorder (DD; nicotine excluded) patients. These are defined as conditions in which abuse of or dependence on substances such as alcohol, cocaine, opioids, phencyclidine, amphetamine, cannabis, or nicotine, negatively impacts on family and social life, work, and school. SUDs are also associated with impairment or distress (even if formal criteria of dependence are not necessarily met), and may causes financial problems (1).

WHO Mental Health surveys have suggested an association between psychotic experiences and SUDs, even if not all types of SUDs are associated with psychotic episodes (2). About 25–50% of psychotic patients are diagnosed with schizophrenia and a concomitant addiction disorder (3). Moreover, the prevalence of SUD is 25.1% in patients with schizophrenia and 20.1% in those with bipolar disorder, with young men affected by schizophrenia having the highest prevalence of non-alcohol drug-use disorder (4). Co-occurrent drug use is a frequent condition among patients presenting with a first episode of psychosis, with a prevalence ranging from 25 to 60% (5). In addition, substance abuse might trigger psychotic symptoms in certain individuals, since substances may be used to self-medicate psychotic symptoms (6, 7). Importantly, DD/psychosis is associated with more frequent psychotic relapses and emergency admissions, and with a tendency for chronicity (8); self-medication may also be related to the presence of psychotic symptoms (9). In this complex setting, adequate treatment is difficult to provide, which is further hampered by the known poor compliance of patients.

Psychotic patients with co-occurrent drug abuse may be more sensitive to the side effects of antipsychotics (i.e., extrapyramidal, cardiovascular, and metabolic) and therefore choosing agents proven with a low liability for such adverse events is warranted (10). Moreover, long acting antipsychotics with the above characteristics may be preferred in this clinical setting both reasons related to adherence and pharmacokinetic considerations (11).

There has been little effort to deliver a common clinical and procedural framework for DD patients and separate policies have focused on either severe mental health problems or addiction (12). At present, the aim of SUD treatment and prevention strategies has changed from a reduction in the use of substances to a greater prevention of the social and clinical consequences of substance use (12). While in some countries mental health services lead treatment programs, in others, such as Italy, addiction services normally care for people with SUD, including those with psychotic episodes. In Italy, indeed, the Drug Addiction Units [Servizi per le tossicodipendenze (SERT); and servizi per le dipendenze (SERD)] have generally been considered autonomous, and separate from Community Psychiatric Services, despite the frequent co-presence of a psychotic disorder and SUD. This has been increasingly recognized as ineffective, and the Departments of Mental Health (DSM) now allow access to users who were heretofore not treated at such centers. In addition, many traditional Therapeutic Communities are also recognizing the difficulty of working with patients with a psychotic disorder and SUD and have begun to adopt the services offered accordingly. In fact, DSM and SERD are increasingly called upon to adopt convergent guidelines and intervention procedures, for an integrated, multi-dimensional, and targeted interventions. This is especially relevant when managing chronic pathologies that require long-term treatment and the use of multiple resources.

In order to shed more light on the current practices for the management of patients with psychotic episodes and concomitant SUD in Italy, we have carried out a survey of psychiatrists on current routine practice among prescribers. Particular emphasis was put on clinical practice in patients suffering from schizophrenia/schizoaffective disorder and substance use (DD/psychosis). Beyond the Italian mental health and Addiction Service system, these aspects could be relevant to identify at-risk patients, improve current prescribing practices, and possibly implement early intervention programs.

## Materials and methods

### General description of survey

This was an *ad hoc* survey consisting of 17 questions intended to investigate the models of care of psychotic patients with concomitant SUD in Italy regarding the prescribing practices of antipsychotic medications in psychotic patients with SUD. The survey was drafted by the authors of this article and made available for completion to psychiatrists throughout the country under the aegis of the Società Italiana Psichiatria delle Dipendenze^1^ The completed surveys were collected in two ways. First, through an electronic poll carried out at the national congress of Società Italiana di Psicopatologia (SOPSI) in 2017, where 98/350 participants responded to the questionnaire (28%). This percentage can be considered to be an adequate representation of participants based on literature data (12–15). In a second *ad hoc*, snowball sampling recruitment technique (in which participants were asked to recruit additional subjects from acquaintances), 5,155 psychiatrist subscribers to the Medikey newsletter (https://ssl.medikey.it/) were asked to fill in the questionnaire, of whom 350 (6.8%) did. Thus, a total of 448 completed questionnaires were collected. Participation in compilation of the questionnaires was entirely voluntary, and no incentive for participation was offered. A link was provided to an online participant information sheet, and upon consenting were participants redirected to the online questionnaire. There were no mandatory responses in the questionnaire and participants were free to withdraw at any stage in compilation, and participants wishing to withdraw could simply close the relevant page. All answers and questionnaires were completely anonymous, and other than the information detailed below no personal identifying information was collected.

The survey included a series of questions on the respondents' demographics (age, sex, specialty, position, type of setting, and geographic location), besides the 17 questions. Of these, questions 1–6 investigated the models of care of psychotic patients with concomitant SUD, questions 7–10 queried about the characteristics of patients seen in daily practice by psychiatrists, and questions 11–17 focused on the prescription practices and long-term formulations employed in the subgroup of patients with schizophrenia/schizoaffective disorder. The translated questionnaire is shown in Data Sheet 1 in Supplementary Material.

## Results

### Demographics of respondents

A total of 448 questionnaires were collected. As summarized in Table 1, 56.5% of the respondents were men and nearly 62% were older than 50 years; >40% responded that they were Head of Department or similar, while only 7.6% were resident fellows.

**Table 1:** Demographics of study respondents.

	**Percentage**
Sex	
Male	56.5
Female	43.5
Age (years)	
<40	19.4
41–50	18.8
51–60	38.0
>60	23.9
Specialty	
Psychiatry	88.8
Neurology	4.7
Other	6.5
Position	
Director of integrated department/center	11.0
Head of simplified structure	20.5
High-level specialist	19.2
Hospital physician^*^	41.7
Resident fellow	7.6
Type of structure	
Hospital structure	20.5
Day hospital services	42.6
Residential setting	7.6
Addiction services	14.7
Private practice	14.5

* *Not general practitioners*.

The type of settings of respondents was fairly well-balanced: indeed, over 40% worked in specialized centers, while the remainder was divided between local structures, private practice and addiction services, besides residential settings. The respondents were from all areas of Italy, although the highest percentages were from Lombardy (16.3%), Lazio (10.9%), Campania (9.4%), and Sicily (9.2%).

### Models of care of psychiatric patients with concomitant SUD (Q1-Q6)

In the Italian practice, comorbid substance abuse was most frequently diagnosed within the context of anxiety disorder (46%), followed by bipolar disorder (25%), and schizophrenia/schizoaffective disorder (12%) (Q1, Table 2). Notably, 84.4% of respondents felt that patient management was becoming more complex due to substance abuse, while the reminder said “no” or “don't know” (Q2). With regard to the most prevalent type of structure for psychiatric patients with comorbid substance abuse in their center (Q3), 49% responded that co-management with addiction services was the optimal approach, followed by management by a psychiatrist within the context of a multidisciplinary group (21%). Only 12% felt that exclusive psychiatric management was the best approach. The clinical choice was overwhelmingly acknowledged as the major driver (48%) in the decision of the model of care, followed by the good working relationship between client services (18%); only 4% said that costs were a major motivator in their choice (Q4). The overall level of integration with other services (Q5) was mostly viewed as good/excellent (16.7%) or acceptable (39.3%). However, 31.9% deemed it as unacceptable. When asked in what context psychiatric patients with comorbid substance abuse are presented (Q6), a wide variety of responses was given: addiction services (27%), specialized center (26%), regional/local services (19%), and emergency services (15%).

**Table 2:** Questions regarding comorbid substance abuse and various treatment centers.

**Question**	**Answers**	% Responders^*^(%)
1. Which of the following conditions are most frequently diagnosed as comorbid at your center?	Schizophrenia/schizoaffective disorder and SUD	12
	Bipolar disorder and SUD	25
	Mood disorders (other) and SUD	7
	Anxiety disorder and SUD	3
	Personality disorders and SUD	46
2. Do you have the impression that your work has become more complex in the last 5 years due to comorbidities?	Yes	84
	No	8
3. What is the prevalent model used for management of patients in your structure?	Management by a psychiatrist alone	12
	Management by a psychiatrist within a multidisciplinary group	21
	Co-management with addiction services	49
	Referred to addiction services	4
	Insertion in a residential structure	3
4. What is the reason for adopting the model chosen?	Clinical choice	48
	Good working relationship between services	18
	Poor working relationship between services	14
	Cost considerations	4
	Insertion in a residential structure	5
5. What is the level of integration with other services?	Poor	32
	Acceptable	39
	Good	15
	Excellent	2
6. What service do you preferentially use as initial treatment for comorbid SUD?	Emergency room	15
	Hospital structure	19
	Day hospital services	26
	Addiction services	27
	Community therapy	0

* *The number of non-responders is not indicated*.

*SUD, substance use disorder*.

### Characteristics of patients with psychotic episodes and SUD (Q7–Q10)

**Figure 1:**
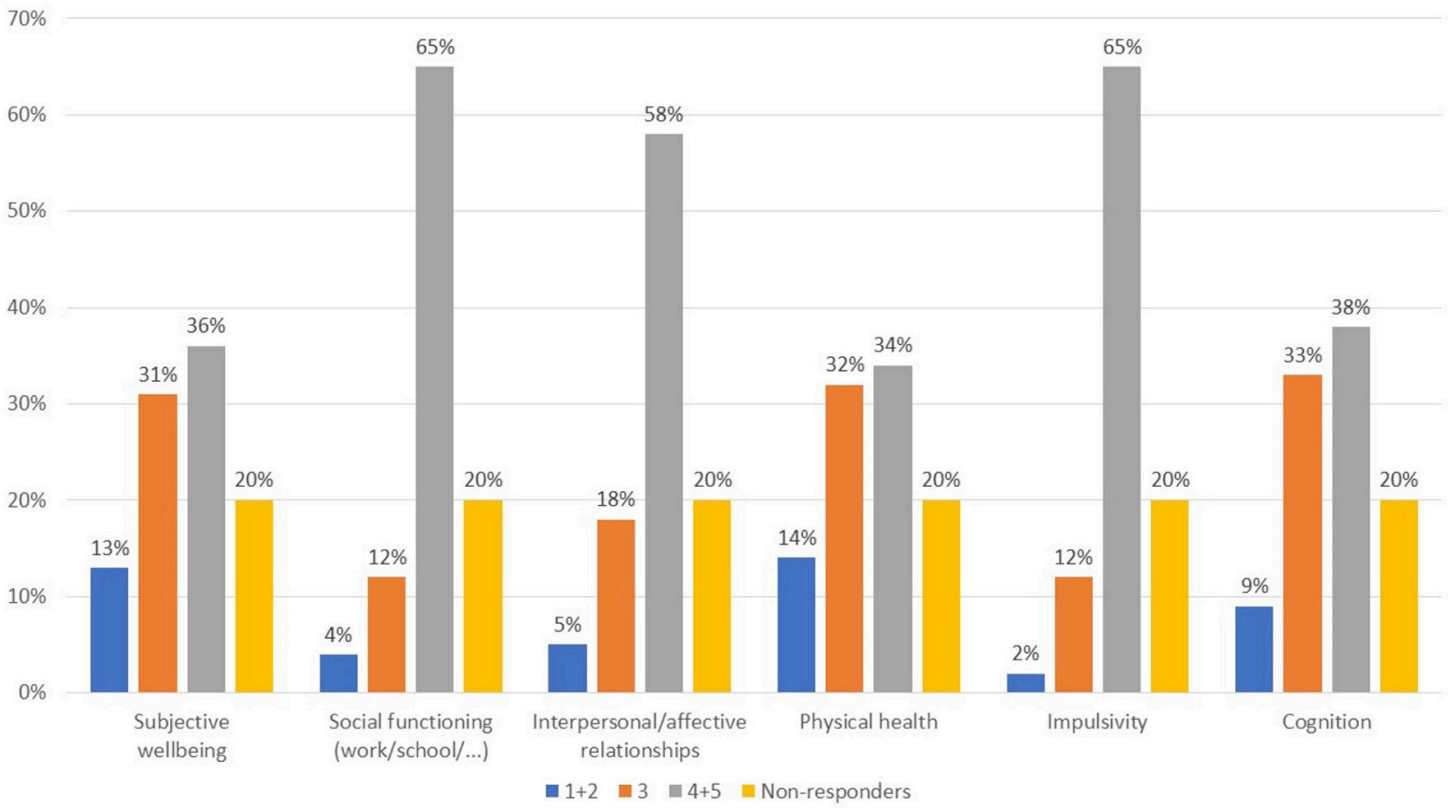
What areas are most compromised in psychotic patients with comorbid substance abuse? Value expressed on a scale of 1–5 where 1, not important; 2, not very important; 3, somewhat important; 4, important; 5, very important.

**Figure 2:**
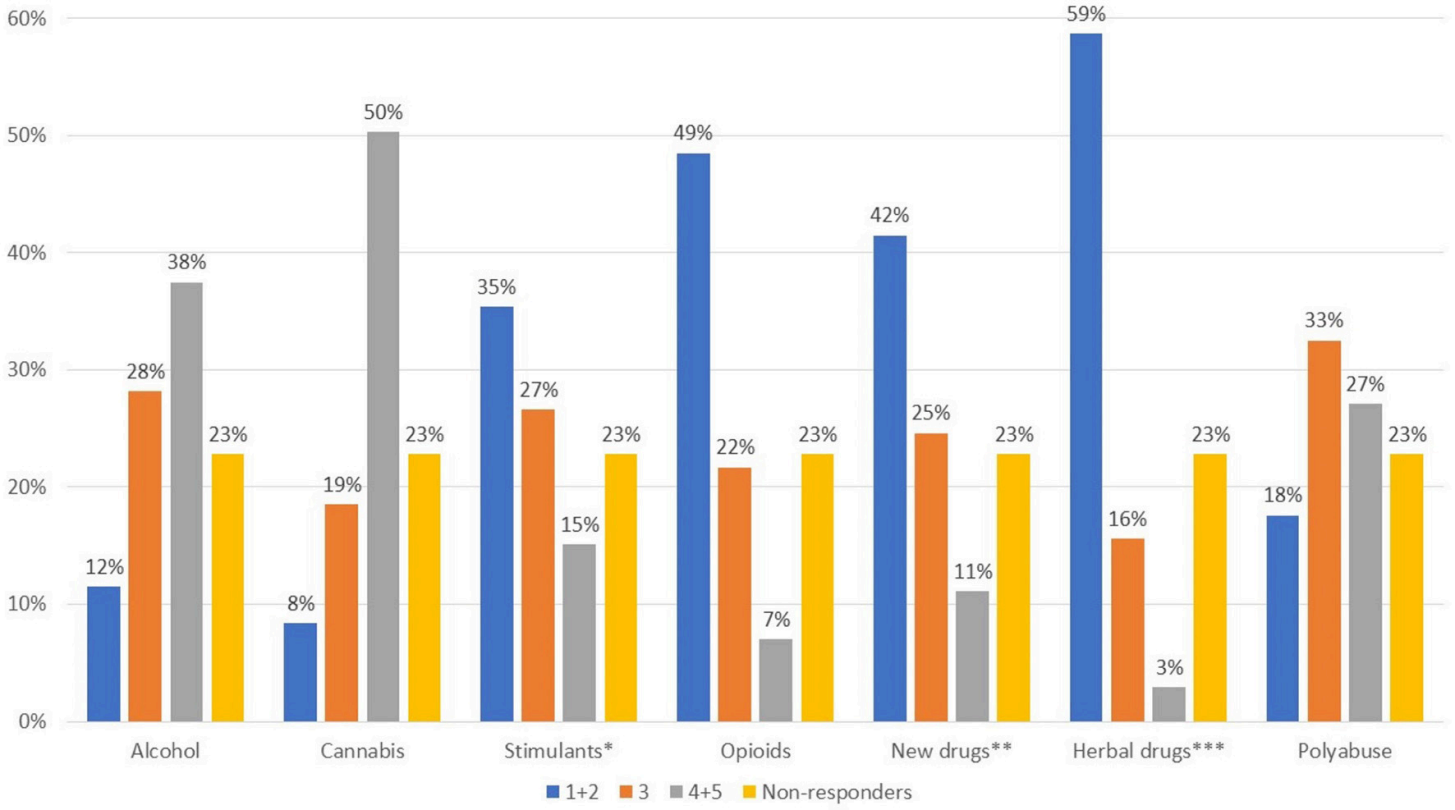
What type of substances are most frequently used by patients with psychotic episodes? Value expressed on a scale of 1–5 where 1, not used; 2, infrequent; 3, somewhat used; 4, often used; 5, very often used *cocaine, amphetamines; ** e.g., mephedrone, synthetic cannabinoids, Spice drugs, latest generation ecstasy derivatives, ketamine, and derivatives; ***hallucinogenic mushrooms, Salvia divinorum, Ayahuasca, Mitragyna speciosa-kratom.

The areas reported to be the most affected in patients with comorbid substance abuse (Figure 1, Q7) were functioning, interpersonal relations, and impulsivity, while more heterogeneous responses were given for the remaining areas. Questions 8 and 9 were optional and are not presented herein as only a small number of responses were collected. With regard to the types of substances used in psychiatric patients, a wide variety of responses were seen (Figure 2, Q10). Cannabis and alcohol were among those most used, although stimulants were also used in more than 40% of patients. Fewer but significant proportions of patients were using opioids or other drugs, and even fewer new synthetic drugs or plant-derived hallucinogens. Of note, about 60% of patients had used more than one substance.

### Patients with schizophrenia/schizoaffective disorder and SUD: prescribing practices and long-term agents (Q11–Q17)

**Figure 3:**
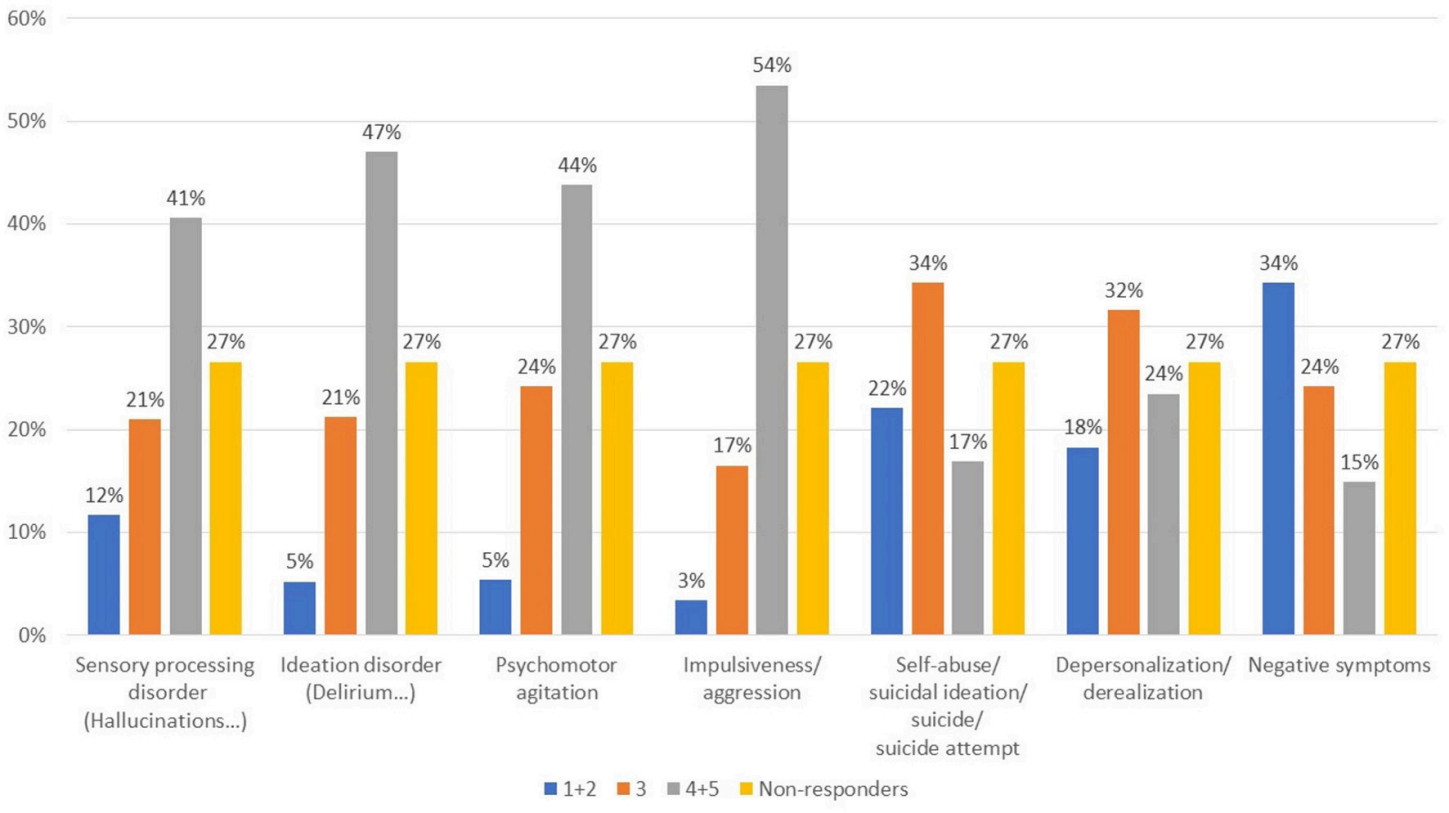
What symptoms are most represented in patients with schizophrenia/schizoaffective disorder and SUD? Value expressed on a scale of 1–5 where 1, rare; 2, infrequent; 3, somewhat frequent; 4, often; 5, very often.

In patients with schizophrenia/schizoaffective disorder and comorbid substance abuse, the symptoms most frequently reported (Figure 3, Q11) were sensory perception disorders, ideation, agitation, and impulsivity. Of note, negative symptoms were predominant in a small proportion of subjects. In their daily practice, 51.9% of respondents used to treat differently this subgroup from other psychiatric patients, whereas 23.2% did not (25.0% did not respond) (Q12).

**Figure 4:**
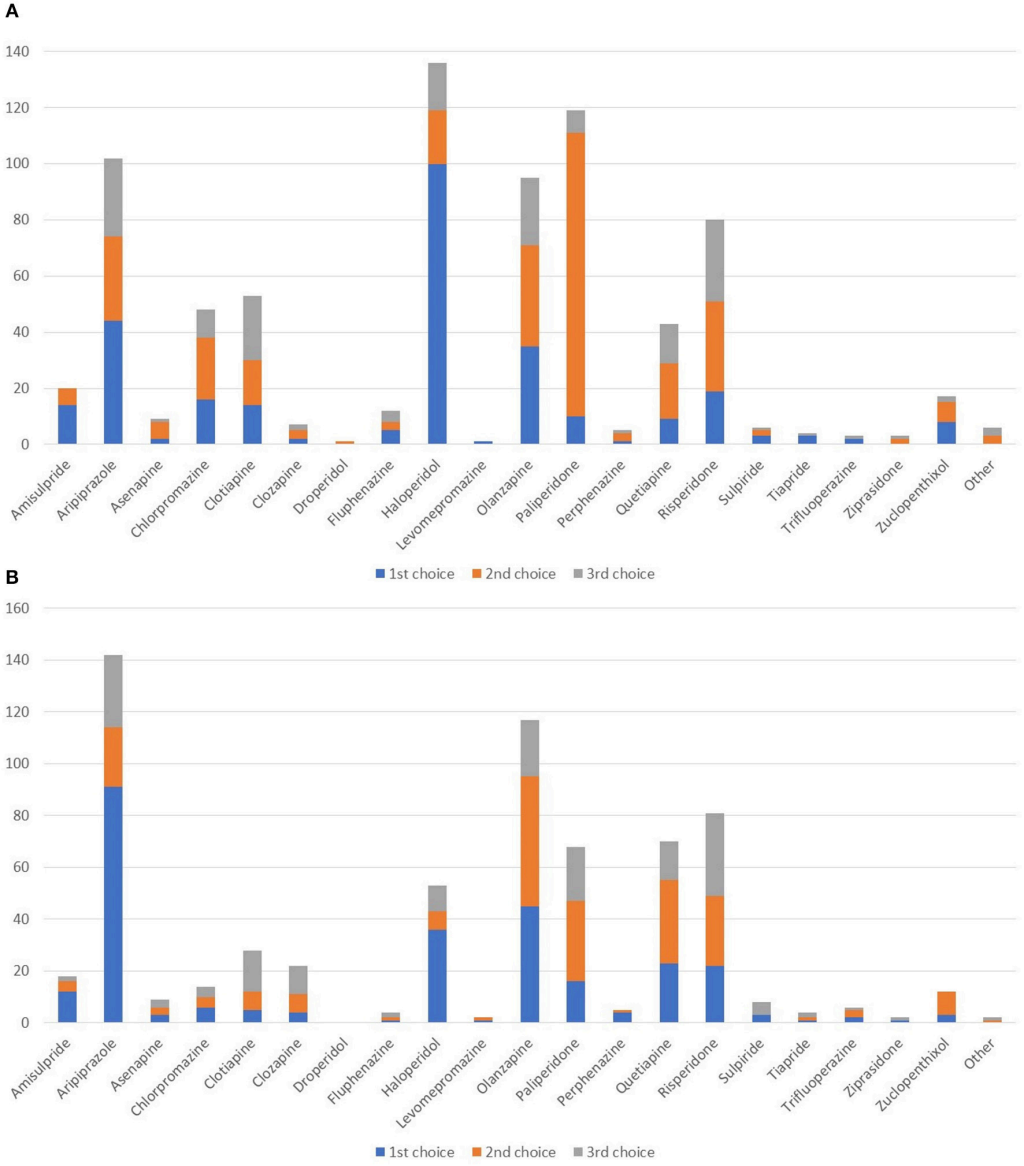
Which agents do you most often prescribe as first-, second-, or third-line in patients with schizophrenia/schizoaffective disorder and SUD in the acute **(A)** and in maintenance **(B)** setting?

In the acute setting (Figure 4A), haloperidol was widely seen as the first-line agent of choice followed by aripiprazole and olanzapine. Paliperidone was the most commonly chosen second-line agent. In the maintenance phase, aripiprazole was the prevalent choice as first-line agent, followed by olanzapine (Figure 4B). Quetiapine and risperidone were also frequently used.

**Figure 5:**
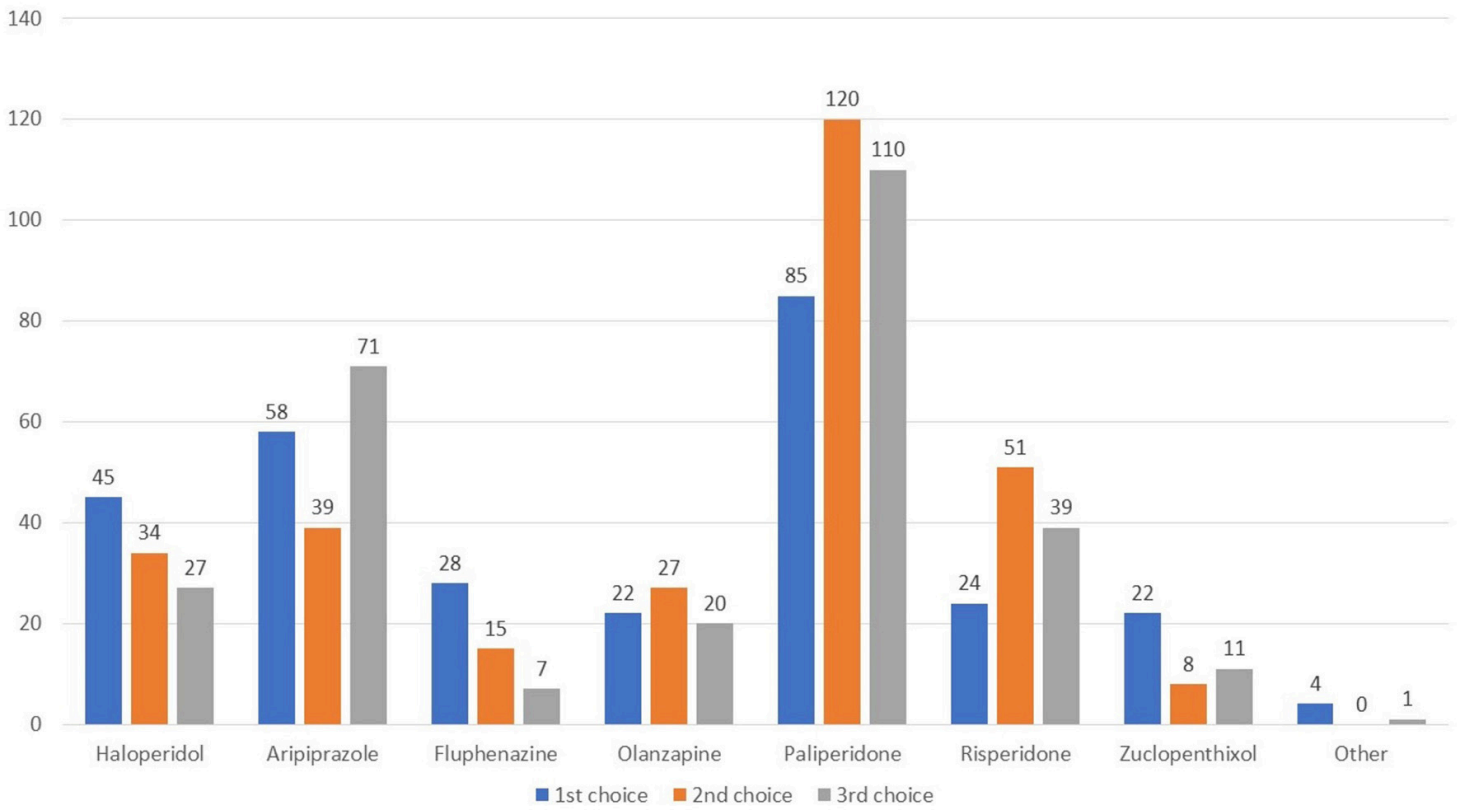
Which long-acting agent do you prefer to prescribe as first, second, and third line therapy?

The remaining questions queried about the use of long-acting agents 48.6% of respondents used long acting agents, while 35.0% did not (Q14). 74.9% prescribed prevalently an atypical agent, while 10.5, 8.2, and 6.4 stated that they usually preferred neither atypical or typical agents, a long-acting acting combined with an oral agent, and a typical agent, respectively. Figure 5 shows the agents rated as first, second, or third choice. Paliperidone was the long-acting agent most widely adopted in all three lines, followed by aripiprazole, haloperidol, and risperidone.

**Figure 6:**
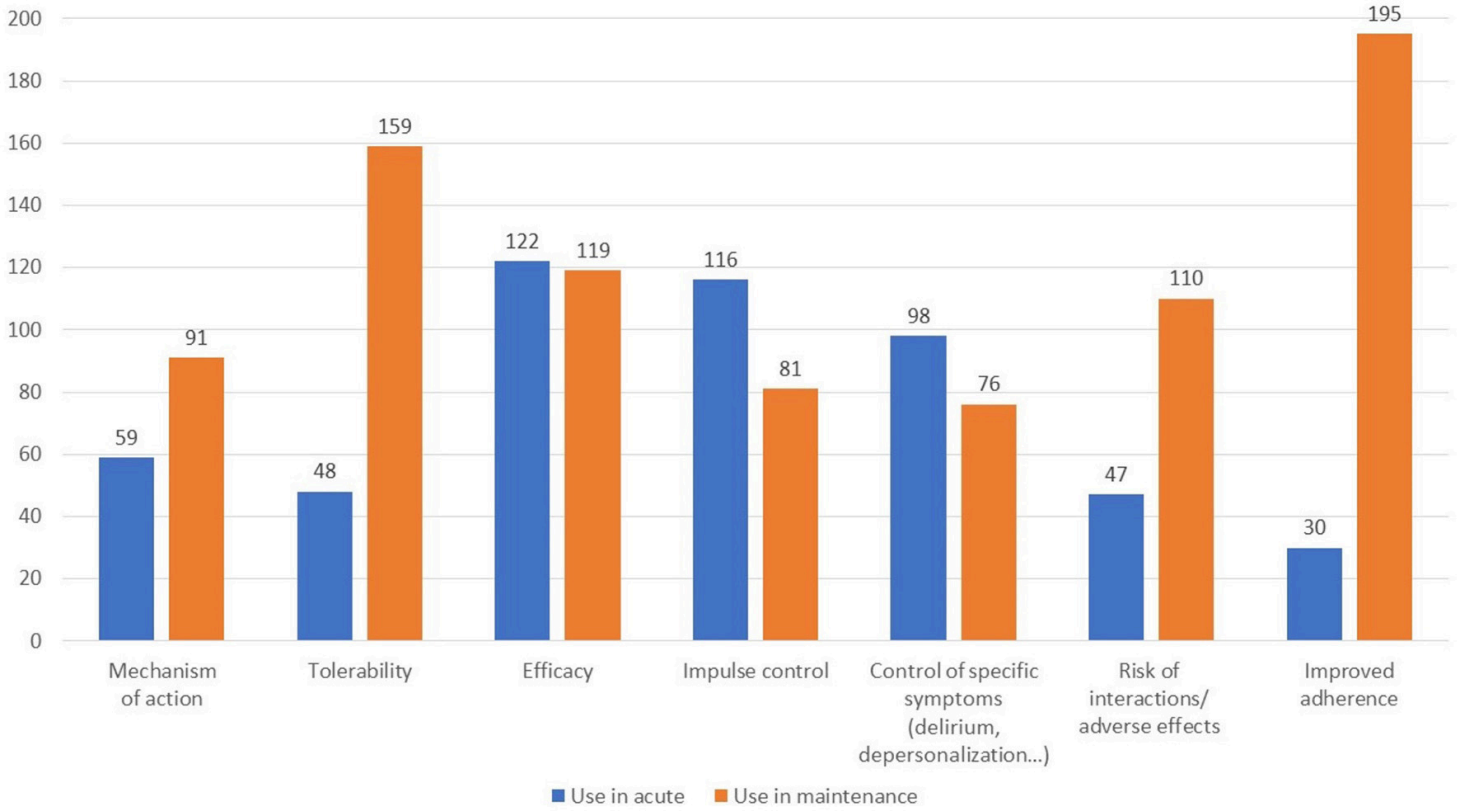
What is the main motivating factor for choice of a long-acting antipsychotic?

**Figure 7:**
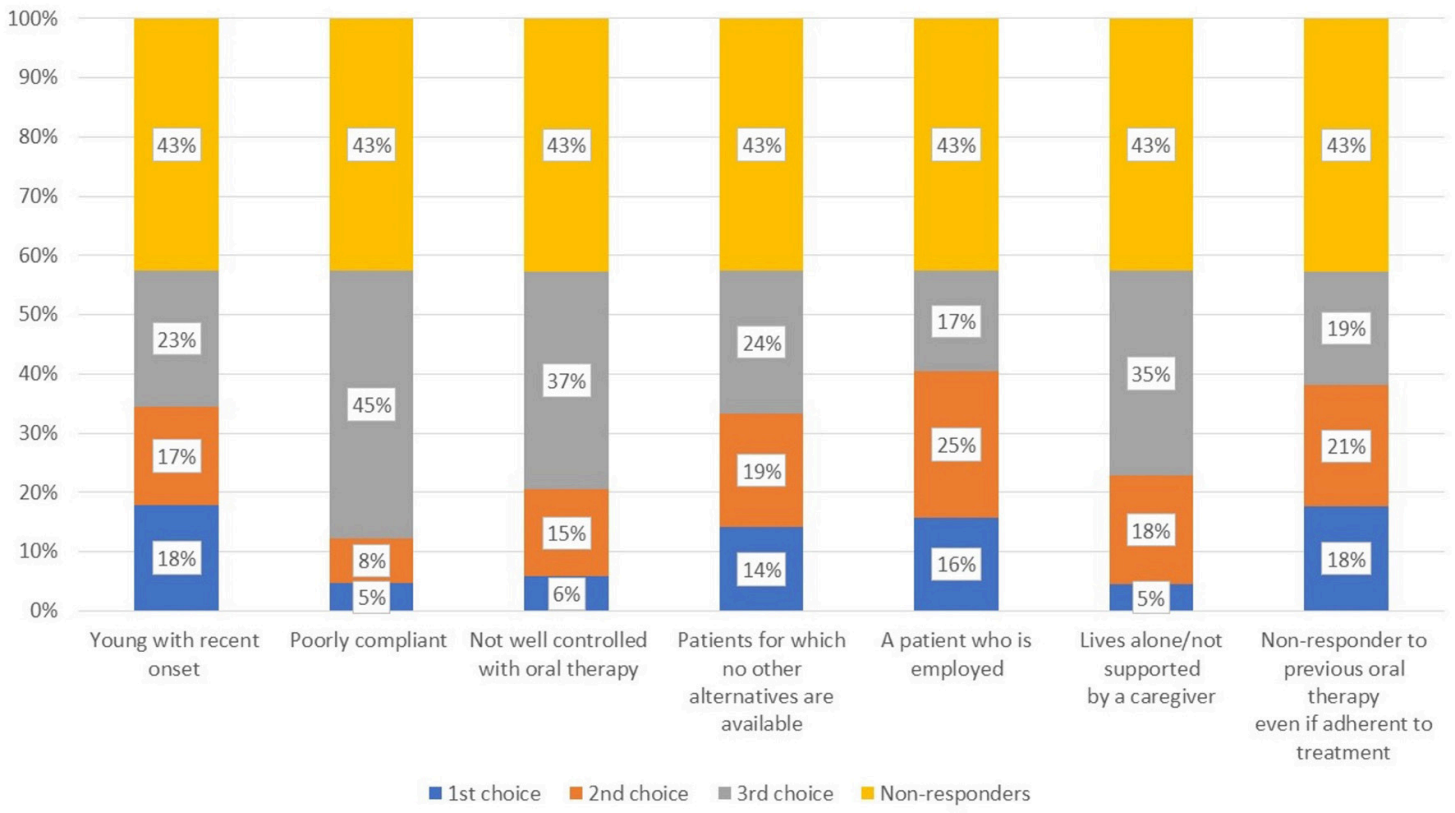
In which type of patient with SUD are long-acting antipsychotics most suited?

When asked about the reason for choosing a long-acting antipsychotic in the acute and maintenance phases (Q15), there was a wide range of responses for the former, most frequently citing efficacy, control of impulsivity, and control of specific symptoms (Figure 6). In the maintenance phase, better adherence and tolerability were the main drivers. Regarding the most suitable patient for a long-acting antipsychotic in those with and without comorbid substance use (Figure 7, Q16), a large proportion of participants did not respond, while many indicated the subjects with poor compliance and poorly controlled by oral therapies. In addition, respondents often cited those living alone or who were not well-supported by a caregiver as good candidates.

**Figure 8:**
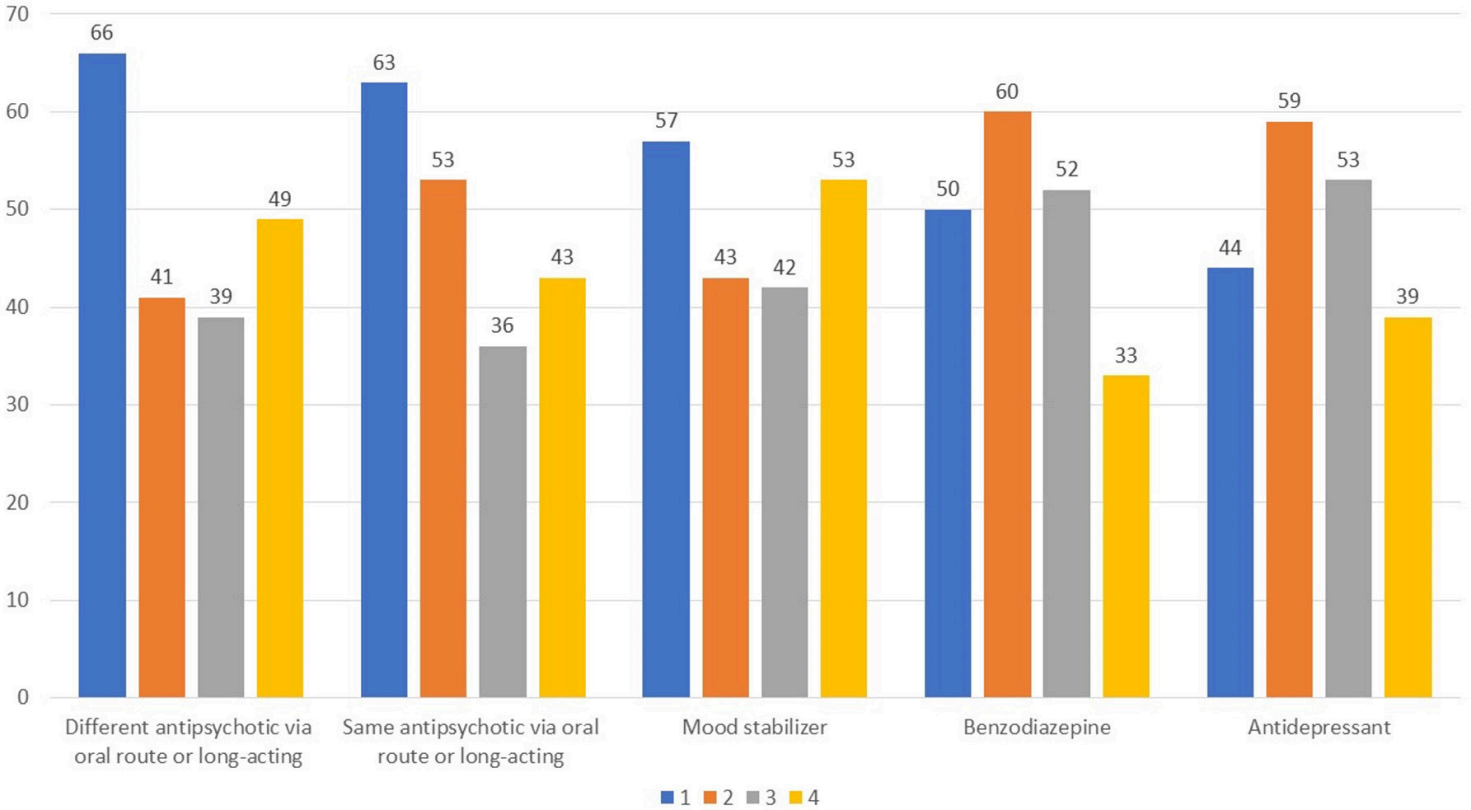
Which types of drugs are most widely used in combination with a long-acting antipsychotic? Value expressed on a scale of 1–4 to estimate the frequency, 1 for most frequent use and 4 for less frequent use.

Finally, according to 43% of respondents the concomitant use of other psychoactive drugs in those being treated with long-acting antipsychotics is frequent, whereas another 43% responded no (Q17). Interestingly, there was little consensus on what types of drugs were more commonly used in concomitance (Figure 8).

## Discussion

The respondents of the present survey were primarily psychiatrists working in a wide range of clinical settings significantly representing real-life practice. Addiction services do not commonly treat psychotic patients with SUD, representing only 14.7% of respondents. Indeed, from the present survey, the majority of the psychotic patients with SUD are treated at outpatient services and in hospital (63.1% combined) in line with what is believed to be the optimal approach, and much fewer in residential settings. In addition, general psychiatrists are likely to be those who formulate a diagnosis of SUD and psychosis, and prescribers may not always have adequate training on diagnosis of a DD.

It was our intention to gain more information about the current prescribing practices for patients with schizophrenia and SUD in Italy. The results of the present survey are consistent with previous studies indicating that SUD is common in patients with schizophrenia. Interestingly, the vast majority of respondents felt that management of comorbid SUD is becoming more complex. Thus, on one hand it is not surprising that many viewed the use of a multidisciplinary team as the optimal approach for such patients. It is also reassuring that costs did not appear to be a major motivator for this choice. As expected, interpersonal relationships and overall functioning were among the areas most seen to be affected by comorbid substance abuse, with patients abusing a variety of substances, most often alcohol and cannabis, but also other drugs. The majority of patients also had poly-substance abuse. The present survey can be seen as an extension of a previous one carried out over a decade ago on DDs in Italy (16).

It is noteworthy that negative symptoms were seen as predominant in only a minority of patients, with other symptoms being more prevalent, such as sensory perception disorders, ideation, agitation, and impulsivity. This could perhaps suggest that the antipsychotic treatment was not optimal in terms of the agent prescribed and/or dosing. However, such additional factors were not queried in the present survey. Nonetheless, in the acute phase haloperidol was often the agent of first choice, followed by the second-line agents aripiprazole and paliperidone. In the maintenance phase, aripiprazole appeared to be the first-line choice, followed by other second-line agents. This finding confirms that second-line agents are indeed widely prescribed in schizophrenic patients with comorbid substance abuse, and that those with lower metabolic impact are also favored. The prescribing preferences referred would also seem to indicate that the choice of agent is not specifically related to the presence of a single diagnosis of psychosis or DD/psychosis. Since reward deficiency syndrome is more easily established in patients with SUD, this suggests that the dopaminergic resources of those with SUD are already destabilized by substance use (16), with the development of anhedonia and/or hypophoria as probably the most relevant clinical symptoms (17). It is now commonly agreed that dopamine is a major neurotransmitter in terms of reward dependence, even if there is some controversy regarding its clinical modulation in treatment and prevention of prevent relapse for SUD. While many have advocated that medications blocking dopamine should be favored, it can also be argued that short-term blockade of D2 receptors is warranted, but that long-term treatment should activate and not completely inhibit D2 receptors in order to enhance the functional connectivity of brain reward circuits (16, 18–23).

Some psychoactive substances and medications can be related to “Reward Deficiency Syndrome,” which was originally described as an outcome of chronic alcohol and stimulant abuse, and a highly relevant topic of which clinicians should be aware within the context of SUD. In part, it links with the a-motivational syndrome (AS) (24), displayed as an expression of chronic cannabis intoxication, but which is closely related to post-withdrawal syndrome (PWS) described by Martin et al. as an enduring pathologic state in abstinent detoxified opiate addicts (25). From a withdrawal-related point of view, through each detoxification cycle the patient passes from the acute withdrawal state (counter-polar to intoxication—psychomotor retardation in case of cocaine acute withdrawal) to a later and enduring drug-free state featuring symptoms of anhedonia or hypophoria, looming as acquired discomfort related to the absence of drug-related stimulation. Hypophoria includes somatic, vegetative (sleep), mood, and anxiety symptoms such as susceptible or irritable (depressed) mood, amplified pain perception, inability to perform simple tasks, and make normal efforts, and inability to experience reward in any way other than substance use. This syndrome closely resembles the subthreshold symptoms of dysthymia and the residual symptoms of chronic bipolar disorder (26, 27). This is one of the possible ways of relapsing behavior.

With regards to the use of long-acting antipsychotics, roughly one-half responded that they use these agents in clinical practice, with clear preference for the use of an atypical agent in this formulation, in line with a recent study recently conducted in Italy (28). In this regard, a study comparing long-acting injectable formulations of aripiprazole and paliperidone in patients with comorbid psychosis and SUD found that both agents improved clinical status and QoL and reduced substance craving at 1 year (29). Among long-acting formulations, paliperidone was widely favored in the present survey. Respondents cited efficacy and better adherence as major motivators for prescription of a long-acting agent in the maintenance phase, along with good tolerability. Patients with poor compliance and those living alone with inadequate caregiver support were believed to be most suited for a long-acting antipsychotic. Lastly, there was little agreement as to which type of drugs were most commonly used in combination with a long-acting antipsychotic. This could be indicative that many different types of drugs are used in conjunction with long-acting agents, including oral antipsychotics, mood stabilizers, benzodiazepines, and antidepressants.

It is difficult to compare the present results with those in the literature as there is limited real-life data on prescribing habits in patients with schizophrenia and comorbid SUD. In a recent survey among French psychiatrists, most psychiatrists used second-generation antipsychotics, and preferentially an oral formulation, in the treatment of schizophrenia (30). Long-acting agents were prescribed in about one-third of schizophrenic patients, and the duration and type of practice did not influence the class or formulation of antipsychotics used. As perhaps can be inferred from the present survey, in that survey personal experience, government regulatory approval, and guidelines for the treatment of schizophrenia were the main factors that guided decision-making. However, that survey was not specific on patients with comorbid substance abuse. In a survey of practice in 7 Central and Eastern European Countries, oral atypical antipsychotics, mostly risperidone, olanzapine, clozapine, were among those most commonly prescribed for schizophrenia (31). As in the present survey, anxiolytics (70%), antidepressants (42%), and mood-stabilizers (27%) were commonly co-prescribed in the survey mentioned.

In previous survey of Italian psychiatrists treating schizophrenia, while efficacy and tolerability were among the most widely used factors used to evaluate treatment outcomes in patients with schizophrenia, overall quality of life and global functioning were also considered to be important. These findings were also mirrored in the present survey. Notwithstanding, some discrepancies in quality of care indicators have been revealed among Italian DSM, with a percentage of inappropriate interventions ranging from 5.9 to 66.8% for pharmacological interventions, with significant variability in monitoring of metabolic effects, psychosocial rehabilitation, family involvement, and work (32). These results underscore the greater need for monitoring of patients and better integration of mental health services, aspects which were also seen in the present survey as inadequate for a proportion of respondents.

In this regard, it is worthwhile to stress that while awareness is definitely increasing, psychiatrists should be vigilant to the adverse metabolic effects of some second-generation antipsychotics (33), especially the risk of reward deficiency syndrome in substance user psychotic patients treated with full blocking D2 antagonist. To improve the current situation, additional efforts are still needed in order to improve training programs as shown in a recent survey of 35 countries (34). In considering the ideal antipsychotic, long-acting agents are considered to be superior to their oral equivalents in reducing relapse, even if current schizophrenia treatment guidelines often give preference to oral formulations (35).

A relevant issue in long term therapy schizophrenia and drug addiction comorbidity is represented by treatment-resistant schizophrenia. Approximately 30% of schizophrenia patients do not respond or respond poorly to antipsychotics treatment (36), treatment resistant schizophrenia is defined as lack of response to at least two different antipsychotics at 600 mg chlorpromazine equivalent and for the duration of treatment of at least 6 weeks. These patients show reduction in milestone acquisition (37), more cognitive impairment (38), and functioning deficits as well as higher incidence of neurological soft signs (39) compared to patients who respond to antipsychotic treatment.

The relationship between treatment resistance and drug abuse is two-fold. Drug abuse or addiction, besides worsening adherence to treatment, can interfere with the efficacy of antipsychotics by worsening symptoms of the disease and/or interacting with the pharmacodynamics of antipsychotics (40). On the other hand, worsening of psychiatric symptoms may be relevant to further induce abuse and/or addiction. Long acting injectable antipsychotics could overcome some of these issues by improving adherence and therefore excluding false positive treatment-resistant patients, as well as possibly increasing the availability of the antipsychotics at the receptor level.

Overcoming these and other barriers in the future will undoubtedly help to optimize management of the difficult-to-treat group of patients with schizophrenia and comorbid substance abuse. From the responses to the present survey, it is clear that the respondents are aware of the problem of SUD in psychotic patients. Not only can treatment be optimized in terms of the choice and formulation of antipsychotics, but greater emphasis should be placed on treatment efficacy, tolerability, and especially the potentially negative metabolic consequences of antipsychotics, with the consequent need for better monitoring of patients. Despite the limitations of the present study, such as possible bias related to the survey techniques used and potential bias in the profile of responders, it seems clear that new treatment paradigms will be needed, and achieving these long-term goals will also require even greater awareness and training. The present survey nonetheless helps to better understand current routine practice among prescribers.

## Author contributions

All authors contributed to the design of the study, analyzed and interpreted the results, revised the manuscript in each section and approved the final version.

### Conflict of interest statement

The authors declare that the research was conducted in the absence of any commercial or financial relationships that could be construed as a potential conflict of interest.

## Footnotes

^1^http://www.sipdip.it/documenti/Report%20finale%20PADDI%20Study.pdf

## Supplementary material

Data Sheet 1 Questionnaire administered.
