# Supplementary material for: Patterns of Management of Patients With Dual Disorder (Psychosis) in Italy: A Survey of Psychiatrists and Other Physicians Focusing on Clinical Practice
Source: Front Psychiatry. 2018 Nov 13;9:575. doi: 10.3389/fpsyt.2018.00575 (PMC6243108; doi:10.3389/fpsyt.2018.00575)
Supplement: Data Sheet 1 — Questionnaire administered. [file Data_Sheet_1.docx]

NAME *(Optional)*…………………………………………………………………….

TELEPHONE *(Optional)* …………………………………………………………………….

**Age**

< 40

41-50

51-60

> 60

**Sex** F M

**Specialization**

**a.** Psychiatry

**b.** Neurology

**c.** Other (specify)

**POSITION**

**a.** Director of integrated department/center

**b.** Head of simplified structure

**c.** High-level specialist

**d.** Hospital physician

**e.** Resident fellow

**f.** Other _________________

**TYPE OF STRUCTURE:**

**a.** Hospital

**b.** Day hospital

**c.** Residential setting

**d.** Addiction services

**e.** Private practice

**REGION**

Val d’Aosta 1 Marche 11

Piemonte 2 Lazio 12

Lombardia 3 Abruzzo 13

Liguria 4 Molise 14

Veneto 5 Campania 15

Trentino-Alto Adige 6 Puglia 16

Friuli-Venezia Giulia 7 Basilicata 17

Emilia-Romagna 8 Calabria 18

Toscana 9 Sicilia 19

Umbria 10 Sardegna 20

**1. Which of the following conditions are most frequently diagnosed as comorbid at your center?**

**a.** Schizophrenia/schizoaffective disorder and SUD

**b.** Bipolar disorder and SUD

**c.** Mood disorder (other) and SUD

**d.** Anxiety disorder and SUD

**e.** Personality disorder and SUD

**2. Do you have the impression that your work has become more complex in the last 5 years due to comorbidities?**

**a.** Yes

**b.** No

**3. What is the prevalent model used for management of patients in your structure?**

**a.** Management by a psychiatrist alone

**b.** Management by a psychiatrist within a multidisciplinary group

**c.** Co-management with addiction services

**d.** Referred to addiction services

**e.** Insertion in a residential structure

**4. What is the reason for adopting the model chosen?**

**a.** Clinical choice

**b.** Good working relationship between services

**c.** Poor working relationship between services

**d.** Cost considerations

**e.** Insertion in a residential structure

**5. What is the level of integration with other services?**

**a.** Poor

**b.** Acceptable

**c.** Good

**d.** Excellent

**6. What service do you preferentially use as initial treatment for comorbid SUD?**

**a.** Emergency room

**b.** Hospital structure

**c.** Day hospital

**d.** Addiction services

**e.** Community therapy

**7. What functional areas are most compromised in psychotic patients with comorbid substance abuse?**

*On a scale of 1 to 5 where 1 = not important, 2 = not very important, 3 = somewhat important, 4 = important, 5 = very important*

| **a.** Subjective wellbeing | 1 | 2 | 3 | 4 | 5 |
| --- | --- | --- | --- | --- | --- |
| **b.** Social functioning (work/school/...) | 1 | 2 | 3 | 4 | 5 |
| **c.** Interpersonal/affective relationships | 1 | 2 | 3 | 4 | 5 |
| **d.** Physical health | 1 | 2 | 3 | 4 | 5 |
| **e.** Impulsivity | 1 | 2 | 3 | 4 | 5 |
| **f.** Cognition | 1 | 2 | 3 | 4 | 5 |

**8. Which of the following tools do you know/use to evaluate comorbidities?**

|  | **KNOW** | **USE** |
| --- | --- | --- |
| AUDIT |  |  |
| ASI |  |  |
| CIWA |  |  |
| VAS |  |  |
| DSM 5/ICD-9 |  |  |
| Other |  |  |

**9. In your experience, what percentage of patients with psychotic spectrum disorders present with use of substances and/or alcohol?**

|  | **SUBSTANCE** | **ALCOHOL** |
| --- | --- | --- |
| 0 - 10% |  |  |
| 10 - 30% |  |  |
| 30 - 60% |  |  |
| > 60% |  |  |

**10. What types of substances are most frequently used by patients with psychotic episodes?**

*On a scale of 1 to 5 where 1 = not used, 2 = infrequent, 3 = somewhat used, 4 = often used, 5 = very often used*

| **a.** Alcohol | 1 | 2 | 3 | 4 | 5 |
| --- | --- | --- | --- | --- | --- |
| **b.** Cannabis | 1 | 2 | 3 | 4 | 5 |
| **c.** Stimulants (cocaine, amphetamines) | 1 | 2 | 3 | 4 | 5 |
| **d.** Opioids | 1 | 2 | 3 | 4 | 5 |
| **e.** New drugs (e.g.: mephedrone, synthetic cannabinoids-Spice drugs, latest generation ecstasy derivatives, ketamine and derivatives) | 1 | 2 | 3 | 4 | 5 |
| **f.** Herbal drugs (hallucinogenic mushrooms, Salvia divinorum, Ayahuasca, Mitragyna speciosa-kratom) | 1 | 2 | 3 | 4 | 5 |
| **g.** Polyabuse | 1 | 2 | 3 | 4 | 5 |

**11. Which of the following psychiatric symptom areas are most frequent in patients with schizophrenia/schizoaffective disorder and SUD?**

*On a scale of 1 to 5 where 1 = rare, 2 = infrequent, 3 = somewhat frequent, 4 = often, 5 = very often*

| **a.** Sensory processing disorder (Hallucinations…) | 1 | 2 | 3 | 4 | 5 |
| --- | --- | --- | --- | --- | --- |
| **b.** Ideation disorder (Delirium…) | 1 | 2 | 3 | 4 | 5 |
| **c.** Psychomotor agitation | 1 | 2 | 3 | 4 | 5 |
| **d.** Impulsiveness/aggression | 1 | 2 | 3 | 4 | 5 |
| **e.** Self-abuse/suicidal ideation/suicide/suicide attempt | 1 | 2 | 3 | 4 | 5 |
| **f.** Depersonalization/derealization | 1 | 2 | 3 | 4 | 5 |
| **g.** Negative symptoms | 1 | 2 | 3 | 4 | 5 |

**12. Do you treat patients with schizophrenia/schizoaffective disorder and SUD differently from your other patients?**

**a.** Yes

**b.** No

**13. Which of the following do you prescribe most often in patients with schizophrenia/schizoaffective disorder and SUD?**

|  | **ACUTE PHASE** | **MAINTENANCE PHASE** |
| --- | --- | --- |
| **a.** Mostly typicals |  |  |
| **b**. Mostly atypicals |  |  |
| **c.** Typical and atypicals in combination |  |  |

**Which of the following do you prescribe most often?**

| **DRUG** | **USE IN ACUTE** | | | **USE IN MAINTENANCE** | | |
| --- | --- | --- | --- | --- | --- | --- |
|  | **FREQUENCY** | | | **FREQUENCY** | | |
|  | **1** | **2** | **3** | **1** | **2** | **3** |
| Haloperidol |  |  |  |  |  |  |
| [Amisulpride](https://it.wikipedia.org/wiki/Amisulpride) |  |  |  |  |  |  |
| [Aripiprazole](https://it.wikipedia.org/wiki/Aripiprazolo) |  |  |  |  |  |  |
| [Asenapine](https://it.wikipedia.org/w/index.php?title=Asenapina&action=edit&redlink=1) |  |  |  |  |  |  |
| Chlorpromazine |  |  |  |  |  |  |
| [Clotiapine](https://it.wikipedia.org/wiki/Clotiapina) |  |  |  |  |  |  |
| Clozapine |  |  |  |  |  |  |
| Droperidol |  |  |  |  |  |  |
| Fluphenazine |  |  |  |  |  |  |
| Levomepromazine |  |  |  |  |  |  |
| [Olanzapine](https://it.wikipedia.org/wiki/Olanzapina) |  |  |  |  |  |  |
| [Paliperidone](https://it.wikipedia.org/w/index.php?title=Paliperidone&action=edit&redlink=1) |  |  |  |  |  |  |
| Perphenazine |  |  |  |  |  |  |
| [Quetiapine](https://it.wikipedia.org/wiki/Quetiapina) |  |  |  |  |  |  |
| [Risperidone](https://it.wikipedia.org/wiki/Risperidone) |  |  |  |  |  |  |
| [Sulpiride](https://it.wikipedia.org/wiki/Sulpiride) |  |  |  |  |  |  |
| [Tiapride](https://it.wikipedia.org/w/index.php?title=Tiapride&action=edit&redlink=1) |  |  |  |  |  |  |
| Trifluoperazine |  |  |  |  |  |  |
| [Ziprasidone](https://it.wikipedia.org/wiki/Ziprasidone) |  |  |  |  |  |  |
| Zuclopenthixol |  |  |  |  |  |  |
| Other |  |  |  |  |  |  |

**14. Do you prescribe a long-acting antipsychotic when treating a patient with schizophrenia/ schizoaffective disorder?**

**a.** Yes

**b.** No

**If yes, which?**

**a.** Mostly typicals

**b.** Mostly atypicals

**c.** No preference for typical or atypical

**d.** long-acting in association with an oral drug

**Among the long-acting drugs, which do you prescribe most often?**

| **DRUG** | **FREQUENCY** | | |
| --- | --- | --- | --- |
|  | **1** | **2** | **3** |
| Haloperidol |  |  |  |
| [Aripiprazole](https://it.wikipedia.org/wiki/Aripiprazolo) |  |  |  |
| Fluphenazine |  |  |  |
| [Olanzapine](https://it.wikipedia.org/wiki/Olanzapina) |  |  |  |
| [Paliperidone](https://it.wikipedia.org/w/index.php?title=Paliperidone&action=edit&redlink=1) |  |  |  |
| [Risperidone](https://it.wikipedia.org/wiki/Risperidone) |  |  |  |
| Zuclopenthixol |  |  |  |
| Other |  |  |  |

**15. Which of the following are most considered when choosing a long-acting antipsychotic?**

|  | **USE IN ACUTE** | **USE IN MAINTENANCE** |
| --- | --- | --- |
| Mechanism of action |  |  |
| Tolerability |  |  |
| Efficacy |  |  |
| Impulse control |  |  |
| Control of specific symptoms (delirium, depersonalization…) |  |  |
| Risk of interactions/adverse effects |  |  |
| Improved adherence |  |  |

**16.** **Please indicate your views with regards to the following statements from a previous survey regarding patients with schizophrenia/schizoaffective disorder and long-acting drugs:**

*Indicate from 1 to 3 where 1 = disagreement, 2 = neutral, 3 = agreement*

| **THE PATIENT MOST INDICATED FOR A LONG-ACTING AGENT IS:** | **WITH SUBSTANCE ABUSE** | | | **WITH NO SUBSTANCE ABUSE** | | |
| --- | --- | --- | --- | --- | --- | --- |
| **a.** young with recent onset | 1 | 2 | 3 | 1 | 2 | 3 |
| **b.** poorly compliant | 1 | 2 | 3 | 1 | 2 | 3 |
| **c.** not well controlled with oral therapy | 1 | 2 | 3 | 1 | 2 | 3 |
| **d.** patients for which no other alternatives are available | 1 | 2 | 3 | 1 | 2 | 3 |
| **e.** a patient who is employed | 1 | 2 | 3 | 1 | 2 | 3 |
| **f.** lives alone/not supported by a caregiver | 1 | 2 | 3 | 1 | 2 | 3 |
| **g.** non-responder to previous oral therapy even if adherent to treatment | 1 | 2 | 3 | 1 | 2 | 3 |

**17.**  **Is the concomitant use of a long-acting antipsychotic and other psychotropic drugs frequent in patients with schizophrenia/schizoaffective disorder and SUD frequent?**

**a.** Yes

**b.** No

**If yes, which of the following classes of drugs are most often used in polythe4rapy with a long-acting antipsychotic?**

*Please indicate from 1 to 4 to estimate the frequency, 1 for most frequent use and 4 for less frequent use.*

| **a.** Different antipsychotic via oral route or long-acting | 1 | 2 | 3 | 4 |
| --- | --- | --- | --- | --- |
| **b.** Same antipsychotic via oral route or long-acting | 1 | 2 | 3 | 4 |
| **c.** Mood stabilizer | 1 | 2 | 3 | 4 |
| **d.** Benzodiazepine | 1 | 2 | 3 | 4 |
| **e.** Antidepressant | 1 | 2 | 3 | 4 |
